# The Effects of the Al and Zr Contents on the Microstructure Evolution of Light-Weight Al_x_NbTiVZr_y_ High Entropy Alloy

**DOI:** 10.3390/ma16247581

**Published:** 2023-12-09

**Authors:** Hongwei Yan, Rui Liu, Shenglong Li, Yong’an Zhang, Wei Xiao, Boyu Xue, Baiqing Xiong, Xiwu Li, Zhihui Li

**Affiliations:** 1 State Key Laboratory of Nonferrous Metals and Processes, China GRINM Group Co., Ltd., Beijing 100088, China; yanhongwei@grinm.com (H.Y.);; 2 GRIMAT Engineering Institute Co., Ltd., Beijing 101407, China; 3 General Research Institute for Nonferrous Metals, Beijing 100088, China

**Keywords:** series of AlNbTiVZr HEAs, element content variation, microstructure evolution, ZrAlV Laves phase

## Abstract

To investigate the comprehensive effects of the Al and Zr element contents on the microstructure evolution of the AlNbTiVZr series light-weight refractory high entropy alloys (HEAs), five samples were studied. Samples with different compositions were designated Al_1.5_NbTiVZr, Al_1.5_NbTiVZr_0.5_, AlNbTiVZr, AlNbTiVZr_0.5_, and Al_0.5_NbTiVZr_0.5_. The results demonstrated that the actual density of the studied HEA samples ranged from 5.291 to 5.826 g·cm^−3^. The microstructure of these HEAs contains a solid solution phase with a BCC structure and a Laves phase. The Laves phase was further identified as the ZrAlV intermetallic compound by TEM observations. The microstructure of the AlNbTiVZr series HEAs was affected by both the Al and Zr element contents, whereas the Zr element showed a more dominant effect due to Zr atoms occupying the core position of the ZrAlV Laves phase (C14 structure). Therefore, the as-cast Al_0.5_NbTiVZr_0.5_ sample exhibits the best room temperature compression property with a compression strength (σ_p_) of 1783 MPa and an engineering strain of 28.8% due to having the lowest ZrAlV intermetallic compound area fraction (0.7%), as characterized by the EBSD technique.

## 1. Introduction

High entropy alloys (HEAs) were preferentially defined by Yeh in 2004 as alloys containing at least five principal elements, each having atomic percentage of between 5% and 35% [1,2]. HEAs are distinct from conventional alloys, which typically contain one or two elements as the so-called solvent. Recent studies suggest that alloys that consist of four principal elements can also be classified as HEAs [3,4,5]. The cocktail effect leads to unpredictable collaborative mixture results, and it is one of the four core effects of HEAs. This provides an innovative approach to the design of complex alloys with exceptional properties including a high mechanical strength, high temperature resistance, corrosion resistance, and radiation resistance properties compared with traditional alloys [6,7,8,9]. Especially, HEAs consisting of refractory elements are often considered to be potential structural materials for static structural parts under high-temperature service conditions and could replace the traditional Ni-based superalloy, which acts as the stabilization structure under high-temperature service conditions, owing to the sluggish diffusion effect.

Generally, refractory elements refer to metal elements with a melting point exceeding 1650 °C, including W, Mo, Nb, V, Ta, and Zr. HEAs containing refractory elements have drawn the interest of researchers due to their intrinsically good high-temperature mechanical properties. Senkov [10], who invented the VNbMoTaW HEAs, which retain a yield strength of 477 MPa, even at 1600 °C, is the pioneer in research on refractory HEAs. The NbTiVZr series HEAs were also first developed by Senkov in 2013. This series of alloys exhibits a yield strength of 834 MPa at 600 °C, while the density is 6.52 g·cm^−3^, making them almost 50% lighter than the VNbMoTaW HEAs [11]. Liao [12] conducted a thorough investigation of the NbTiVZr HEAs’ crystal structures by combining virtual crystal approximation, CALPHAD modeling, and the quasi-harmonic Debye–Grüneisen model and concluded that the BCC structure is the most stable crystal structure compared to the FCC and HCP structures. The elemental composition is a crucial factor that affects the microstructure and properties of HEAs. Comparing the as-cast NbTiVZr_0.5_ alloy to the NbTiVZr and NbTiVZr_2_ alloys, which are fabricated by the vacuum arc melting (VAM) method, shows that an increase in the Zr element content leads to additional Laves phases other than the single BCC structure solid solution phase, as confirmed by XRD tests [13]. To further decrease the density of refractory HEAs, aluminum is introduced into the NbTiVZr series HEAs due to its low density, and the actual density of the Al_x_NbTiVZr (x = 0.5, 1, 1.5) alloys ranges from 5.5 to 6.04 g·cm^−3^ [14]. However, the phase constitution of these AlxNbTiVZr HEAs becomes more complex because of the bond formation ability resulting from the highly negative mixing enthalpy between Al and other refractory metal elements. And, the existence of the Laves phase obviously weakens their plasticity compared to the NbTiVZr HEAs with a single BCC structure. Compression tests showed that the Al_1.5_NbTiVZr alloy fractures before reaching the yield stage. By means of density functional theory, Qiu et al. [15] revealed that the Al-Zr bond contributes to the structural stability of ordered configurations for the AlNbVTiZr alloys by defining the formation heat. Therefore, some studies have focused on the effect of the Zr element content on the structural and mechanical properties of the AlNbTiVZr series HEAs. With a decreased Zr content, the AlNbTiVZr_0.5_ alloy showed an exceptional yield strength of 1430 MPa at room temperature, and only surface cracks were found. Even the cylindrical sample was compressed to half of its height. The presence of some individual Zr_2_Al-typed Laves phase particles observed inside the BCC grain could contribute to its good strength–ductility balance [16]. However, with a further reduction in the Zr content, the compression strain of AlNbTiVZr_0.25_ HEA declined to 6% due to the segregation of Al and Zr elements at the grain boundary and the formation of the Zr_5_Al_3_ phase [17].

Existing research indicates that the excellent properties and ideal microstructure characteristics of the AlNbTiVZr series HEAs are closely related to their compositions. In this work, five samples with different Al and Zr contents were fabricated by the vacuum arc melting process. These samples were designated Al_1.5_NbTiVZr, Al_1.5_NbTiVZr_0.5_, AlNbTiVZr, AlNbTiVZr_0.5_, and Al_0.5_NbTiVZr_0.5_, and the subscripts indicate the atomic ratios. The effects of the Al and Zr element contents on the microstructure evolution and the relevant mechanical properties of these five as-cast light-weight HEAs were investigated in order to screen the optimal Al and Zr composition of AlNbTiVZr series HEAs.

## 2. Experimental Procedure

In this study, the raw materials for HEA preparation were bulk or rod-shaped pure metals with a purity exceeding 99.9%. After removing the surface oxide layer and subsequently ultrasonic oscillating for 5 min, these pure metals were accurately weighed by an electronic balance with a precision of 0.0001 g, which ensured a mass error of ±0.001 g for each principal element. The nominal compositions (at. %) of each light-weight HEA sample are shown in Table 1. The as-cast HEA samples were prepared in a vacuum arc melting oven (DHL400, Sky Technology Development Co., Ltd., Chinese Academy of Science, Shenyang, China), and pure argon was injected as the protection gas to keep the internal pressure at 8 × 10^4^ Pa. A reserved pure titanium ingot was melted to absorb the remaining oxygen, and then the samples were melted and mechanically turned over repeatedly seven times in a copper crucible to make sure that each principal element was evenly distributed.

Initially, cubic samples with lengths of 10 mm underwent electrical discharge wire cutting from the central part of the as-cast HEA ingots. Then, these cubic samples were ground with SiC paper ranging from 800# to 3000# and polished with diamond paste for 10 min. Subsequently, the actual density of each HEA sample was measured three times by the Archimedes method according to GB/T 1423-1996. The samples underwent X-ray diffraction detection (XRD, Ultima IV, Rigaku Corporation, Tokyo, Japan) analysis with a RIGAKU diffractometer and Cu Ka radiation to figure out the phase compositions and structures. The raw XRD data were subjected to the Rietveld method and subsequently calibrated in Jade 6.5 software according to formerly published studies and relevant PDF database cards. A scanning electron microscope (SEM, JEOL JSM 7001F, JEOL Ltd., Tokyo, Japan) equipped with electron backscatter diffraction (EBSD) and an energy dispersive spectroscopy (EDS) detector was used to observe the microstructure, identify the phase content, and determine the element distribution for these light-weight HEA samples. Furthermore, disk-shape samples, 3 mm in diameter, were cut and mechanically thinned to 60 μm. These samples were twin-jet electropolished with 9% HClO_4_-methanol (vol. %) solution at −15 °C and 30–35 V in preparation for transmission electron microscope (TEM, JEM-2010, JEOL Ltd., Tokyo, Japan) observations. EBSD tests were also performed in the stress-free zone near to the light transmission area of the TEM samples. The compression test is a widely used method to evaluate the mechanical performance of materials. Thus, cylindrical HEA samples with diameters of 6 mm and heights of 9 mm underwent compression tests three times at a speed of 1 mm·min^−1^ using the universal testing machine (MTS 858, MTS Systems, Minnesota, USA) according to ASTM D695.

## 3. Results and Discussion

### 3.1. Density of AlNbTiVZr HEAs

Density is a fundamental physical attribute of materials that represents the extent of the space occupied and is closely related to the atomic mass and lattice arrangement of a substance. Considering that these light-weight HEAs are disordered solid solutions, the nominal density (*ρ*) of each sample was calculated using Equation (1).

(1) $$\rho =\frac{\sum c_{i}A_{i}}{\sum \frac{c_{i}A}{\rho_{i}}}$$

where *c_i_*, *A_i_*, and *ρ_i_* are the atomic percentage, atomic weight, and density of the *i*th principal element of these HEA samples, respectively. As shown in Table 1, the actual density of these samples is below 6 g·cm^−3^ and is consistent with the nominal density. Sample Al_1.5_-Zr_0.5_ had the lowest density of 5.291 g·cm^−3^. The addition of the Al element may reduce the actual density of AlNbTiVZr series HEAs, because aluminum is the lightest element of the five principal elements.

### 3.2. Structure of AlNbTiVZr HEAs

**Figure 1:**
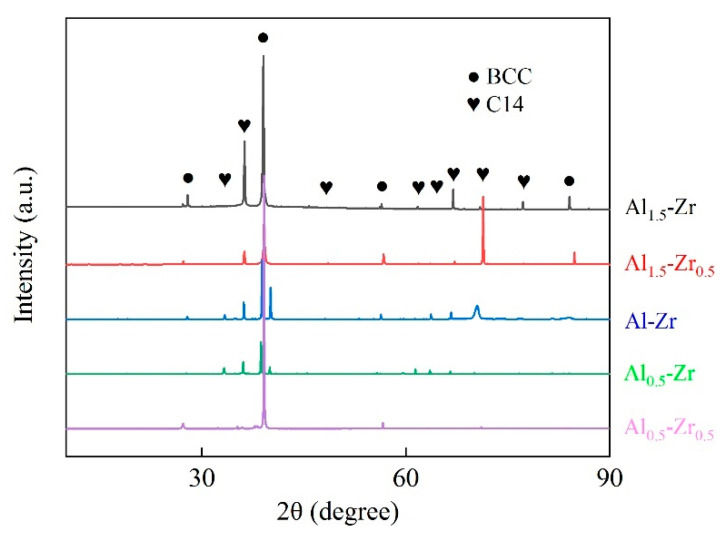
XRD results of AlNbTiVZr light-weight HEA samples.

Among the five principal elements, the Nb and V simple substances exist in the single BCC structure throughout their entire solid-state range, whereas the Zr and Ti simple substances transform into BCC phases in high-temperature ranges. The XRD results of as-cast light-weight AlNbTiVZr HEAs are illustrated in Figure 1. The microstructural compositions of these samples are predominantly composed of the ordered BCC phase and C14-Laves phase, which is consistent with prior studies [14]. Meanwhile, some unmarked low intensity diffraction peaks are shown between the peaks of the BCC phase, indicating the presence of Zr_5_Al_3_ precipitation. Compared to the Al_1.5_-Zr_0.5_ and Al_0.5_-Zr_0.5_ samples, the Al_1.5_-Zr and Al_0.5_-Zr samples show an additional C14-Laves phase diffraction peak with an increase in the Zr element content. Regarding the Al element variation, the main diffraction peak intensity for the BCC phase continues to increase for the Al_1.5_-Zr, Al-Zr, and Al_0.5_-Zr samples. In XRD experiments, a higher diffraction peak intensity indicates a better crystallization performance and a more ordered lattice arrangement. The Al_0.5_-Zr_0.5_ sample exhibits the strongest diffraction peak of the BCC structure with the lowest Al and Zr element contents. It is reasonable to suggest that the C14-Laves phase of these AlNbTiVZr alloys is an Al-Zr enriched phase.

**Figure 2:**
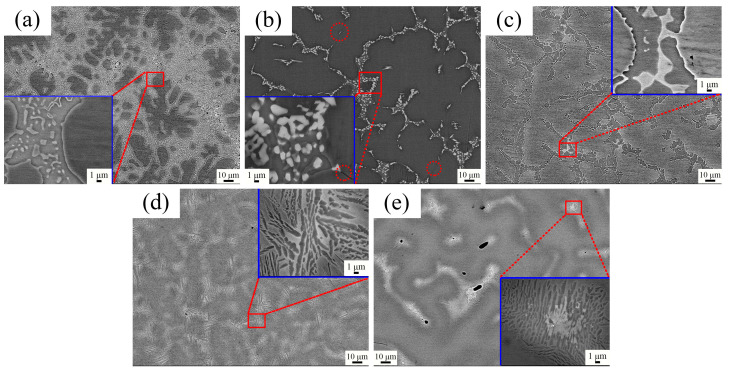
BSE images for AlNbTiVZr HEAs: (**a**) Al_1.5_-Zr of Sample 1; (**b**) Al_1.5_-Zr_0.5_ of Sample 2; (**c**) Al-Zr of Sample 3; (**d**) Al_0.5_-Zr of Sample 4; (**e**) Al_0.5_-Zr_0.5_ of Sample 5.

Figure 2 shows the backscattered electron (BSE) pattern microstructural images of AlNbTiVZr samples. In general, it is notable that the bright phase tends to form near the dark phase boundary, and casting defects such as pores exist inside the bright phase, as shown in the low magnification images. As in the high magnification images, the bright phase shows a heterogeneous composition, which is found to be a thin, elongated, and lens-shaped structure at low Al element contents for the Al_0.5_-Zr and Al_0.5_-Zr_0.5_ samples, while it transforms into a tiny, blocked structure with an increase in the Al element content in the Al_1.5_-Zr sample. As shown in the Figure 1, the Al_1.5_-Zr sample possesses a large amount of bright and cellular-shaped dark phases. In the high magnification image, it is observed that fine, blocked phases are randomly distributed in the region between two dark phases. With a decreasing Zr element content, the bright phase content is lower than that of the Al_1.5_-Zr sample. The average size of the fine, blocked phases increases from approximately 1 μm to 2 μm. However, there are some cracks shown by red circles along the blocked, bright phase, indicating a suboptimal ductility for the Al_1.5_-Zr_0.5_ sample. As for the equiatomic sample Al-Zr, fine, blocked, bright phases bond together as the Al element content decreases. As revealed by the high magnification images in Figure 2d,f, the dark phase and bright phase are mainly distributed side by side. As with the rapid cooling rate of the water-cooled copper crucible in the VAM device, the bright phases in the Al_0.5_-Zr and Al_0.5_-Zr_0.5_ samples present along the heat transfer direction, because the phases nucleated at the solid–liquid interface eventually develop the thin, elongated, lens-shaped morphology with an abundant constitutional supercooling liquid phase region for heat dissipation but no cellular eutectic space or constituent restriction during solidification. However, the thin, elongated, lens-shaped phases tend to be coherent with a low Zr element content for the Al_0.5_-Zr_0.5_ sample. As measured by Image J software (1.53g), the area fraction of the bright phase increases from 35.5% to 52.7% and 61.4% for the Al-Zr and Al_0.5_-Zr samples compared to the Al-Zr_0.5_ sample, respectively. Overall, as the Zr element content increases, the dark phase tends to be separated by the bright phase, and the grain boundary gradually becomes spherical with a cellular structure, indicating that both the Al and Zr elements influence the phase structure of AlNbTiVZr HEAs.

**Figure 3:**
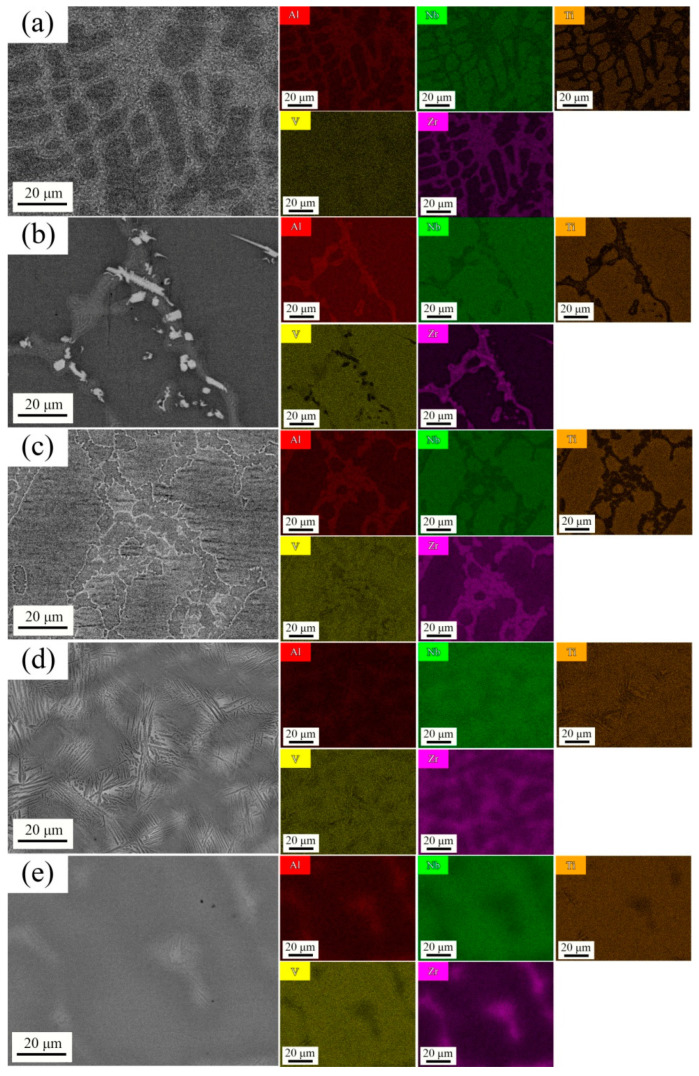
Chemical element distribution images for AlNbTiVZr HEAs: (**a**) Al_1.5_-Zr of Sample 1; (**b**) Al_1.5_-Zr_0.5_ of Sample 2; (**c**) Al-Zr of Sample 3; (**d**) Al_0.5_-Zr of Sample 4; (**e**) Al_0.5_-Zr_0.5_ of Sample 5.

Figure 3 and Table 2 provide insights into the element distribution and corresponding actual chemical element content of each HEA sample. As shown in Figure 3, the attractive interaction between the Al and Zr atoms exhibits the highest affinity among the five principal elements in AlNbTiVZr HEAs, which indicates a greater propensity for solution formation within this specific bond. The distribution of the V element appears to be uniform, suggesting that the C14-Laves phase is associated with the ZrAlV intermetallic. Moreover, some V element voids that are filled with the Al and Zr elements are observed in the mapping image (Figure 3b). Thus, the unmarked peaks in previous XRD tests could be attributed to the presence of Zr_5_Al_3_ precipitation. During the VAM process, Al element evaporation occurs due to the fairly large melting point difference between aluminum and the other refractory elements, resulting in the actual Al element contents of these samples being smaller than the nominal composition. Notably, the Al element differences between the actual and nominal compositions for the Al_0.5_-Zr and Al_0.5_-Zr_0.5_ samples seem smaller than those of other samples. Evaporation induces a local concentration gradient for the bright phase during solidification, leading to thin, elongated, lens-shaped phases in low Al element content samples along the concentration gradient of this Al-rich phase. However, when it provides excess stabilized Al atoms, the thin, elongated, lens-shaped structures disappear in high Al element content samples. On the other hand, Al element evaporation could also bring about casting defects such as pores inside the bright C14-Laves phases. From an energetic point of view, referring to the binary mixing enthalpy (∆*H*_mix_) of the AlNbTiVZr series HEAs in Table 3, the Zr element shows a strong separation tendency from the Nb element, which initially comes into the solidification stage, due to the positive value of ∆*H*_mix_ within the Nb-Zr bond. Meanwhile, the Zr element has negative enthalpy from mixing with the other two elements (V, Al). The positive mixing enthalpy means that the chemical bond suppresses the formation of the ordered configuration, while the negative values favor the formation of an ordered configuration. Hence, during the non-equilibrium solidification caused by the rapid cooling rate of the VAM process, the peritectic reaction is barely carried out completely due to the insufficient diffusion time, and segregation of the Zr and Nb elements and enrichment of the Zr, Al, and V elements occur, which facilitates the high-temperature BCC phase of the Ti element being retained.

### 3.3. Compression Property of AlNbTiVZr HEAs

**Figure 4:**
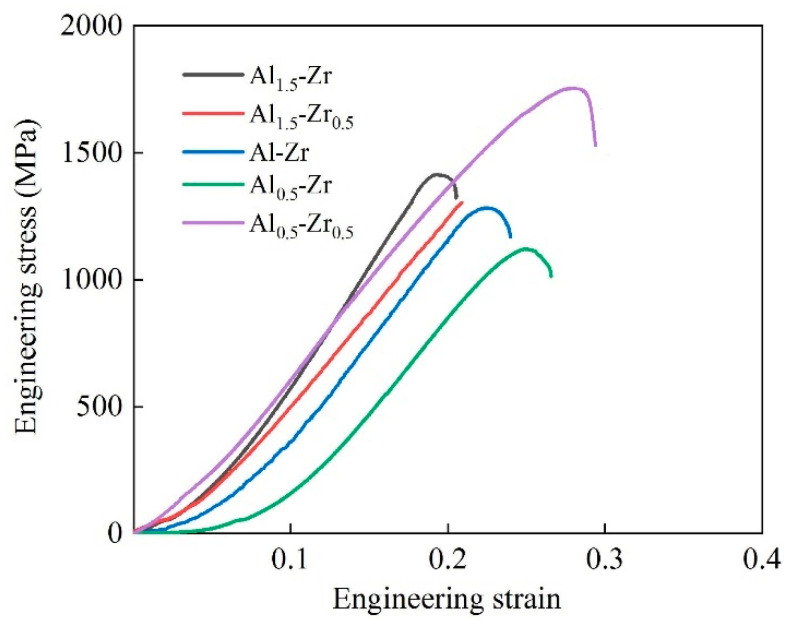
Engineering stress–strain curve for the AlNbTiVZr HEAs.

Figure 4 illustrates the engineering stain–stress curves of all five samples, and each curve was repeatable for the three compression tests conducted. It can be concluded that the HEA samples cannot endure a long yield stage during the compression test, and the Al_0.5_-Zr_0.5_ sample exhibited the most balanced strength–ductile performance with a peak engineering stress (σ_p_) of 1783 MPa and an engineering strain of 28.8%. Especially, the Al_1.5_-Zr_0.5_ sample cracks before reaching the yield stage owing to the casting cracks shown in Figure 2b, marked by the red circles. When the Al element content is decreased, the σ_p_ decreases, while the engineering strain tends to increase. As for changing the Zr element content, with a lower Al element content at the atomic ratio of 0.5, the Al_0.5_-Zr_0.5_ sample tends to be stronger and more ductile. However, no obvious variation in the mechanical properties is observed for samples with higher Al element contents at the atomic ratio of 1.5. It is suggested that both the Al and Zr elements influence the mechanical properties of AlNbTiVZr HEAs.

### 3.4. Phase Composition of AlNbTiVZr HEAs

**Figure 5:**
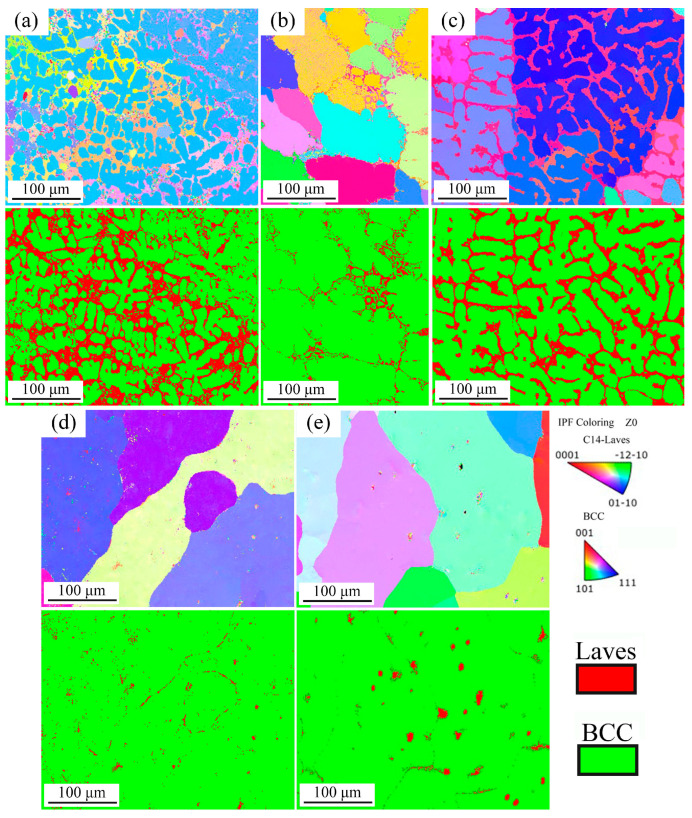
IPF and phase composition images for AlNbTiVZr HEAs: (**a**) Al_1.5_-Zr; (**b**) Al_1.5_-Zr_0.5_; (**c**) Al-Zr; (**d**) Al_0.5_-Zr; (**e**) Al_0.5_-Zr_0.5_.

Based on the EBSD tests, the inverse pole figure (IPF) and phase composition images are shown in Figure 5 by Aztectcrystal software. It can be observed that the BCC phase is the dominant structure in these AlNbTiVZr HEAs, and the Laves phase is extensively distributed on the grain boundary. When the Al element content decreases, the Laves phase content significantly decreases from 29.2% to 23.1% and 1.6% when comparing the Al_1.5_-Zr sample to the Al-Zr and Al_0.5_-Zr samples. Additionally, the width of the Laves phase reduces with a decrease in the Al element content. Figure 5d,e depicts some multicrystalline orientation phases and fine black voids, which are subsequently regarded as Laves phases. For the Al_0.5_-Zr_0.5_ sample with the lowest Al and Zr element contents, the BCC phase content reaches up to 99.3%. However, since both the ZrAlV intermetallic and Zr_5_Al_3_ precipitations have hexagonal structures, this spherical intragranular structure appears to be the Zr_5_Al_3_ precipitation. Consequently, the actual Laves phase content is slightly lower than that shown by the EBSD results in Table 4.

**Figure 6:**
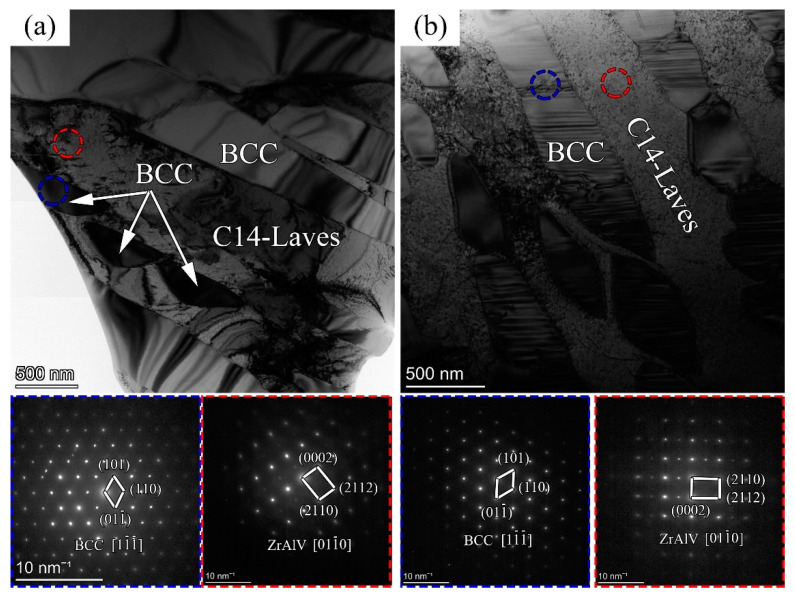
TEM and corresponding SAED pattern images for AlNbTiVZr HEAs: (**a**) Al_0.5_-Zr of Sample 4; (**b**) Al_0.5_-Zr_0.5_ of Sample 5.

As the Al_0.5_-Zr and Al_0.5_-Zr_0.5_ samples were demonstrated to have low Laves phase contents, these samples were further investigated by the TEM technique to elucidate the effect of the Zr element content on the microstructure evolution of AlNbTiVZr HEAs. As shown in Figure 6, the corresponding SAED patterns determined that these alloys consist of the BCC structure phase as the standard electron diffraction pattern along $\left[1\overset{-}{1}\overset{-}{1}\right]$ and the ZrAlV (C14-Laves structure) intermetallic in accordance with the PDF #05-0312 cards. According to the Hume–Rothery rules, an alloy can only form a simple solid solution when its principal elements possess an analogous crystal structure, atomic dimensions, and electrochemical features. However, aluminum is different from the other principal elements in that aluminum obtains a FCC structure, which is unfavorable for the formation of a simple solid solution. The atomic radii of the Zr, Al, and V elements are 160, 143, and 134 pm, respectively. Furthermore, the atomic radius ratio between Zr and (Al, V) is approximately 1.12 to 1.19, which closely aligns with the theoretical optimum nucleation atomic radius ratio (R_A_/R_B_) of 1.225 for the AB_2_ Laves phase (C14 structure). The radius difference between the Zr and (Al, V) atoms results in improved lattice packing and enhances their interactions, ultimately promoting the stability of the ZrAlV Laves phase.

This type of intermetallic with a greater hardness and melting point usually exhibits poor deformability [19,20]. The slip systems of the hexagonal structure ZrAlV phase are prone to activation during the compression process, and then dislocations tend to segregate to the Laves phase boundary in the initial stage of deformation. As the compression process proceeds, the dislocations gradually pile up near the grain boundary, resulting in an intragranular localized stress gradient. This stress gradient can lead to intergranular cracking along the ZrAlV intermetallic, ultimately reducing the compression properties of these samples. As shown in Figure 6, the BCC and C14-Laves structures exhibit a nearly parallel distribution. The average width of the C14-Laves structure reduces from 1 μm to 0.5 μm with a decrease in the Zr element content, validating the previous findings from EBSD tests. The BCC phases are shown as a discontinuous block distribution, separated by the ZrAlV intermetallic. Thus, dislocations require greater kinetic force to slip and accumulate at the sharp Laves phase boundary, resulting in longer elastic deformation stages for the Al_0.5_-Zr_0.5_ sample. In addition, the narrow distance between the BCC phases deteriorates the plasticity, making it prone to fracture upon reaching the yield stage, as proven by the rapid stress reduction during the compression test. Overall, decreasing the Zr content increases the deformability of the AlNbTiVZr series of HEAs at a specific Al element content.

**Figure 7:**
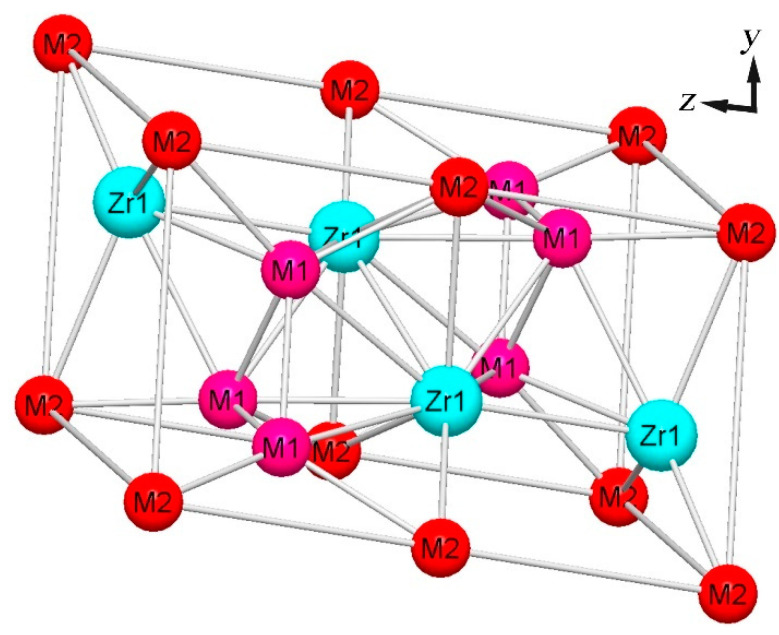
Three-dimensional atomic structure of the ZrAlV intermetallic.

The findings of this study suggest that both the Al and Zr elements significantly affect the intermetallic phase content for AlNbTiVZr light-weight HEAs. And, it is implied that the Zr element has a more dominant influence on the stabilization of the C14-Laves structure (ZrAlV phase). As shown in Figure 7, the three-dimensional structure of the C14-Laves phase observed in the XRD results was reconstructed by CrystalMaker, according to the PDF #05-0312 cards. The Zr atoms occupy the central 4f Wyckoff position, while the Al and V atoms are distributed randomly on the 2a and 6h Wyckoff positions. Considering that the atomic structure of the ZrAlV intermetallic is similar to those of ZrAl_2_ and ZrV_2_ phases, the thermodynamic behavior of this ternary phase is comparable to these binary phases. Generally, when the atomicity of two principal elements approaches the stoichiometric ratio, the formation possibility of the Laves phase increases. According to the ZrAl_2_ and ZrV_2_ binary phase diagram [21,22], it is evident that the solidification temperature range for these binary intermetallic is narrowed with a decline in the Zr element. As a result, numerous Zr-based intermetallics could not coarsen completely, leading to the formation of fine ZrAlV particles, in which the former steady 2a and 6h Wyckoff positions of the binary alloys are replaced by the Al and V atoms. In conclusion, the central Zr atom plays a more dominant role in forming the ZrAlV intermetallic compared to the Al element.

In addition, the bond order is a critical factor in determining the strength of the interatomic bonds. The concept of bond order is introduced in molecular orbital theory, and is equal to half the difference between the number of electrons in the bonding orbital and the number of electrons in the anti-bonding orbital. According to the chemical bond theory in structural chemistry, a larger positive bond order value indicates a stronger and more stable bond between adjacent atoms, making it more difficult to break and thereby increasing the stability of the configuration. Conversely, a negative bond order value indicates an unstable bond between adjacent atoms, which inhibits the stability of the structural configuration. The bond orders between the Zr, Al, and V atoms in the ZrAlV Laves phase and the equiatomic AlNbTiVZr HEA are listed in Table 5. All bond orders between the Zr, Al, and V atoms are positive. The bond order of the V–V bond increases from 0.331 to 0.602, providing a binding kinetic force that promotes phase formation and maintains the structural stability of the ZrAlV intermetallic. Furthermore, the bond orders of the Zr–Al and Zr–V bonds in the Laves phase decrease compared the AlNbTiVZr HEA solid solution, indicating the dominant influence of the Zr element in stabilizing the ZrAlV Laves phase.

## 4. Conclusions

In this study, the comprehensive effects of the Al and Zr element contents on the microstructure evolution and the relevant mechanical properties for AlNbTiVZr series HEAs were examined. The following conclusions are drawn.

1. The actual densities of the studied AlNbTiVZr series HEAs are all below 6 g·cm^−3^, ranging from 5.291 to 5.826 g·cm^−3^. And, these alloys can be classified as light-weight HEAs.
2. These AlNbTiVZr HEAs predominantly exhibit the BCC structure and C14-Laves phase, which was further confirmed to be the ZrAlV intermetallic. Both the Al and Zr element contents influence the microstructure evolution, and the Zr element has a more dominant influence on the phase composition, as the Zr atoms occupy the central position in the ZrAlV Laves phase. The Laves phase content notably decreases from 29.2% to 23.1% and 1.6% for the Al_1.5_-Zr sample compared to the Al-Zr and Al_0.5_-Zr samples which have lower Al element contents. The width of the Laves phase decreases as the Al element content becomes lower.
3. The brittle Laves phase is prone to aggregation and separation of the BCC structure, and it significantly influences the compression mechanical properties of the AlNbTiVZr HEAs. Remarkably, the Al_0.5_-Zr_0.5_ sample exhibits the best compression mechanical properties with a compression strength (σ_p_) of 1783 MPa and an engineering strain of 28.8%, due to having the lowest ZrAlV Laves phase content (0.7%). In contrast, the Al_1.5_-Zr_0.5_ sample fails to reach the yield stage as a consequence of solidification cracks induced by its elevated Al element content.

## Figures and Tables

**Table 1:** Detailed element compositions and densities of light-weight AlNbTiVZr HEAs.

Sample	Alloy (*abbr.*)	Nominal Density/g·cm^−3^	Actual Density/g·cm^−3^
1	Al_1.5_NbTiVZr (Al_1.5_-Zr)	5.482	5.337
2	Al_1.5_NbTiVZr_0.5_ (Al_1.5_-Zr_0.5_)	5.345	5.291
3	AlNbTiVZr (Al-Zr)	5.739	5.599
4	Al_0.5_NbTiVZr (Al_0.5_-Zr)	6.049	5.870
5	Al_0.5_NbTiVZr_0.5_ (Al_0.5_-Zr_0.5_)	5.975	5.826

**Table 2:** Actual chemical element compositions of the AlNbTiVZr HEA samples. (at.%).

Alloy	Al	Nb	Ti	V	Zr
Al_1.5_-Zr	22	25	15	17	21
Al_1.5_-Zr_0.5_	25	25	17	19	14
Al-Zr	16	27	16	18	23
Al_0.5_-Zr	10	30	17	19	25
Al_0.5_-Zr_0.5_	11	31	30	22	17

**Table 3:** Binary mixing enthalpy (∆*H*_mix_) for the series of AlNbTiVZr HEAs [18].

∆*H*_mix_ (kJ·mol^−1^)	Al	Nb	Ti	V	Zr
Al	-	−18	−30	−16	−44
Nb	-	-	2	−1	4
Ti	-	-	-	−2	0
V	-	-	-	-	−4

**Table 4:** Phase contents of the light-weight HEAs.

Phase Content (%)	Al_1.5_-Zr	Al_1.5_-Zr_0.5_	Al-Zr	Al_0.5_-Zr	Al_0.5_-Zr_0.5_
BCC	70.8	93.9	76.9	98.4	99.3
Laves	29.2	6.1	23.1	1.6	0.7

**Table 5:** Average bond orders of the ZrAlV Laves phase and the AlNbTiVZr alloy.

BO_average_	Zr-Zr	Al-Al	V-V	Zr-V	Zr-Al	Al-V
ZrAlV Laves phase	0.227	0.285	0.602	0.228	0.217	0.360
AlNbTiVZr alloy	0.354	0.236	0.331	0.273	0.243	0.255

## Data Availability

Data are contained within the article.
